# Delta-9-Tetrahydrocannabinol-Induced Dopamine Release as a Function of Psychosis Risk: ^18^F-Fallypride Positron Emission Tomography Study

**DOI:** 10.1371/journal.pone.0070378

**Published:** 2013-07-25

**Authors:** Rebecca Kuepper, Jenny Ceccarini, Johan Lataster, Jim van Os, Marinus van Kroonenburgh, Joop M. A. van Gerven, Machteld Marcelis, Koen Van Laere, Cécile Henquet

**Affiliations:** 1 Department of Psychiatry and Psychology, South Limburg Mental Health Research and Teaching Network, EURON, Maastricht University, Maastricht, The Netherlands; 2 PsyQ Heerlen, Mondriaan, South Limburg, The Netherlands; 3 Division of Nuclear Medicine, University Hospital and Catholic University, Leuven, Belgium; 4 Faculty of Psychology, Open University of the Netherlands, Heerlen, The Netherlands; 5 Department of Psychosis Studies, Institute of Psychiatry, King’s Health Partners, King’s College London, London, United Kingdom; 6 Division of Nuclear Medicine, Maastricht University Medical Center, Maastricht, The Netherlands; 7 Centre for Human Drug Research, Leiden, The Netherlands; University of Granada, Spain

## Abstract

Cannabis use is associated with psychosis, particularly in those with expression of, or vulnerability for, psychotic illness. The biological underpinnings of these differential associations, however, remain largely unknown. We used Positron Emission Tomography and ^18^F-fallypride to test the hypothesis that genetic risk for psychosis is expressed by differential induction of dopamine release by Δ^9^-THC (delta-9-tetrahydrocannabinol, the main psychoactive ingredient of cannabis). In a single dynamic PET scanning session, striatal dopamine release after pulmonary administration of Δ^9^-THC was measured in 9 healthy cannabis users (average risk psychotic disorder), 8 patients with psychotic disorder (high risk psychotic disorder) and 7 un-related first-degree relatives (intermediate risk psychotic disorder). PET data were analyzed applying the linear extension of the simplified reference region model (LSRRM), which accounts for time-dependent changes in ^18^F-fallypride displacement. Voxel-based statistical maps, representing specific D_2/3_ binding changes, were computed to localize areas with increased ligand displacement after Δ^9^-THC administration, reflecting dopamine release. While Δ^9^-THC was not associated with dopamine release in the control group, significant ligand displacement induced by Δ^9^-THC in striatal subregions, indicative of dopamine release, was detected in both patients and relatives. This was most pronounced in caudate nucleus. This is the first study to demonstrate differential sensitivity to Δ^9^-THC in terms of increased endogenous dopamine release in individuals at risk for psychosis.

## Introduction

The use of cannabis has long been associated with an increased risk of developing psychotic symptoms in healthy individuals, and with poor outcome in patients with psychotic disorder [1], [2]. Furthermore, patients with psychotic disorder as well as individuals at risk for psychosis seem to express increased sensitivity to cannabis [3], [4], [5], [6], [7], [8]. Yet very little is known about the biological underpinnings of this association [9].

Long-term heavy cannabis use, in particular when started during early adolescence, is associated with structural brain changes such as impaired structural integrity of the corpus callosum [10], alterations in white and gray matter [11], and decreased hippocampal and amygdala volumes [12]. However, it has been argued that the use of cannabis is unlikely to increase the risk of psychosis by mechanisms that manifest themselves as major structural brain changes. Alternatively, neurochemical interactions between cannabis and neurotransmitters such as dopamine (DA) may constitute a biological link between cannabis and psychosis [9].

In the human brain, delta-9-tetrahydrocannabinol (Δ^9^-THC, the main psychoactive constituent of cannabis) activates the type 1 cannabinoid receptor (CB1R), one of the most abundant expressed G-coupled protein receptors in the brain [13]. Activation of CB1Rs pre-synaptically inhibits release of neurotransmitters, including γ-aminobutyric acid (GABA), glutamate and DA [14]. DA is thought to play a role in schizophrenia pathophysiology [15] and animal studies suggest that Δ^9^-THC affects DA neurotransmission in several regions of the brain including prefrontal cortex (PFC) and mesolimbic regions [16], [17]. However, direct evidence for interaction between Δ^9^-THC and DA in the human brain to date remains scarce. First insights came from a single case report study with Single Photon Emission Computed Tomography (SPECT) and ^123^I-IBZM. In this study, a 20% decrease in striatal D_2_ receptor binding ratio was observed, indicating increased synaptic DA release, in a medication-free patient with schizophrenia just after using cannabis [18]. Three subsequent studies used neurochemical brain imaging to examine the effects of Δ^9^-THC on DA release in healthy human volunteers. Bossong and colleagues [19] included seven healthy male recreational cannabis users and investigated the effects of Δ^9^-THC on DA release with Positron Emission Tomography (PET) and ^11^C-raclopride. The authors observed small (around 3.5%) but significant decreases in D_2_ receptor binding in two subregions of the striatum, the ventral striatum and the precommissural dorsal putamen after Δ^9^-THC inhalation [19]. The PET study by Stokes and colleagues [20] failed to find significant changes in D_2_ receptor binding after oral administration of Δ^9^-THC in thirteen healthy male volunteers. Similarly, Barkus and colleagues [21] did not observe DA release after intravenous Δ^9^-THC studied with SPET and ^123^I-IBZM.

Notably, patients with psychotic disorder have been shown to be more sensitive to the behavioral and cognitive effects of cannabis [3] and individual differences in sensitivity to cannabis seem to be in part mediated by genetic risk for psychotic disorder, siblings displaying more sensitivity than well controls in a recent large prospective study [6]. Yet, the biological mechanisms underlying differential sensitivity to cannabis associated with genetic risk for psychosis remain elusive, as existing imaging studies have focused on healthy controls with minimal exposure to cannabis. Therefore, the current study measured Δ^9^-THC-induced DA release using PET and the high affinity D_2/3_ radioligand ^18^F-fallypride in a group of healthy cannabis users and, for the first time, two groups with demonstrated increased sensitivity to Δ^9^-THC: patients with psychotic disorder and unrelated unaffected first-degree relatives. We hypothesized that increased sensitivity to cannabis associated with psychosis risk would be expressed by greater induction of endogenous DA release by Δ^9^-THC.

## Materials and Methods

The study was carried out in accordance with the World Medical Association’s declaration of Helsinki and approved by the standing ethics committee of Maastricht University Medical Center. During screening for in- and exclusion criteria, subjects received all information about the different aspects of the study (including information about study content and procedure, and potential risks and benefits), both in oral and written form. Subjects were given time for consideration of about one week. Written informed consent was obtained from all participants. To ensure capability to consent, patients must not be in an acute phase of their illness as based on the judgment of a board-certified psychiatrist. Only participants were included who were capable to consent. Potential participants who declined to participate or otherwise did not participate were not disadvantaged in any other way by not participating in the study.

### Participants

A total of 30 volunteers (10 patients with psychotic disorder, 10 first-degree unrelated relatives of patients with psychotic disorder, and 10 healthy controls) agreed to participate in the study. Participants were recruited through flyers in local coffee shops (cafes where cannabis is sold and consumed legally), newspaper advertisements and through in- and outpatient mental health service facilities in South Limburg, The Netherlands. Inclusion criteria were i) age 18–60 years, ii) sufficient command of the Dutch language, iii) no intellectual impairment (i.e. IQ >80) as ensured by the Dutch version of the Wechsler Adult Intelligence Scale [22], iv) having smoked cannabis at least once in the past 12 months, v) patients only: a diagnosis of psychotic disorder according to the Diagnostic and Statistical Manual of Mental Disorders (DSM-IV) [23], and vi) relatives only: having a first degree relative with a diagnosis of psychotic disorder. Exclusion criteria were i) head trauma with loss of consciousness or neurological disorder, ii) endocrine or cardiovascular disorder, iii) a positive family history of psychotic disorder (controls only), iv) current use of psychotropic medication, v) current use of illicit drugs other than cannabis, vi) current use of alcohol in excess of 5 standard units per day, vii) presence of metal elements in the body, viii) pregnancy or lactation, and ix) a history of claustrophobia.

In addition to the above described in- and exclusion criteria, participants were asked to abstain from cannabis at least 5 days prior to the testing session [24] and from caffeine and nicotine 4 hours prior to the testing session. Urinalysis was carried out to verify drug abstinence (Multipanel Urine Test 6DS1 for amphetamines, methamphetamines, cocaine, opiates, benzodiazepines and cannabis, SureScreen Diagnostics Ltd.). A pregnancy test was done to rule out pregnancy in the female participants. Additionally, abstinence from recent use of alcohol was assured by means of a breathalyzer. Upon arrival, participants received a standardized meal and a caffeine-free beverage. At the end of the experimental procedure, blood pressure and heart rate was measured. All participants stayed under psychological observation until the acute effects of the Δ^9^-THC had faded and it was safe for the participants to return home.

### Baseline Clinical Measures

Diagnoses in the patient group were confirmed using the Operational Criteria Checklist and associated OPCRIT computer program [25]. Presence and severity of psychotic symptoms during the past two weeks was assessed in all participants with the Positive and Negative Syndrome Scale (PANSS) [26]. Cannabis and other drug use in the past 12 months was assessed using the appropriate sections of the WHO Composite International Diagnostic Interview [27] and the Structured Clinical Interview for DSM Disorders [28].

### Δ^9^-THC Preparation and Administration

Preparation and administration of Δ^9^-THC was performed according to Zuurman and colleagues [29]. Δ^9^-THC was purified from *Cannabis sativa* by Farmalyse BV, Zaandam, The Netherlands, in agreement with GMP guidelines, and was dissolved in 200 µl 100 vol% alcohol. The solvent was used as placebo. Drugs were administered by means of a vaporizer (Volcano®, Storz-Bickel GmbH, Tuttlingen, Germany), a technology designed to safely and effectively deliver Δ^9^-THC while avoiding the respiratory hazards of smoking [30]. Approximately 5 minutes before administration, Δ^9^-THC and placebo, respectively, was vaporized and stored in an opaque polythene bag equipped with a valved mouthpiece preventing the loss of Δ^9^-THC in-between inhalations. As practiced at the beginning of the testing session, subjects were instructed to inhale the volume of the bag in 3–5 subsequent inhalations, holding their breath for 10 seconds after each inhalation and without speaking during the inhalation process. Participants received 8 mg of Δ^9^-THC and placebo in a single-blind manner: participants first received the vaporized vehicle as placebo, followed by the active drug, but, in order to avoid expectation bias, were told that the order of administration was random.

### Blood Sampling

Venous blood samples were taken at baseline and 5, 10, 15, and 75 minutes after Δ^9^-THC administration to determine plasma concentrations of Δ^9^-THC and its two main metabolites 11-OH-THC and 11-nor-9-carboxy-THC, as indicated by Zuurman and colleagues [29]. To prevent un-blinding of participants, sham samples were taken at baseline and 5, 10, 15 and 75 minutes after placebo administration.

### Behavioral Measures

For experimental validation a total of 13 Visual Analogue Scales (VAS) [31] were applied inside the scanner repeatedly to assess subjective changes in perception induced by Δ^9^-THC. These included measures of *feeling ‘high’* (1 scale), *external perception* (5 scales) and *internal perception* (7 scales).

### Radiotracer Preparation

The fluorinated substituted benzamide ^18^F-fallypride is a high-affinity antagonist radiotracer used to visualize and estimate both striatal and extrastriatal D_2/3_ receptors [32], [33]. The precursor for tracer synthesis was obtained from ABX (Radeberg, Germany) and labeling was performed on-site using a Raytest Synchrom R&D synthesis module (Raytest, Straubenhardt, Germany). The final product was obtained after reverse-phase high performance liquid chromatographic (HLPC) purification using a Waters XTerra™ RP18 5 µm 7.8 mm×150 mm column and sodium acetate 0.05 M pH5.5/ethanol 70∶30 V/V as mobile phase at a flow rate of 1.5 ml/min. The ^18^F-fallypride eluted after 18 minutes. The collected peak (2 ml) was diluted with 8 ml of NaCl 0.9% and sterile filtered over a Millipore Cathivex-GS 0.22 µm filter. The final product of the radioligand was administered as a sterile solution of 7 mM sodium acetate buffer pH 5.5, 0.72% and 6% ethanol. The specific radioactivity at the time of injection was greater than 37 GBq/µmol (1000 Ci/mmol). Radiochemical purity was >95%.

### PET Data Acquisition and Processing

Participants underwent a single dynamic PET scanning session after intravenous ^18^F-fallypride administration. PET emission was performed conform the one-day PET imaging protocol for ^18^F-fallypride described and used previously by Christian and colleagues [34] and successfully applied by previous studies [35], [36]. This design, in combination with 18F-fallypride, was chosen because it requires only a single radiochemical synthesis and administration, thereby avoiding session effects and minimizing the amount of radiation exposure as well as the overall burden for participants. The latter was of particular importance since patients with psychotic disorder and co-morbid cannabis use constitute a vulnerable and rather difficult study population and reducing the number of sessions was assumed to increase the feasibility of the study in this particular population.

**Figure 1:**
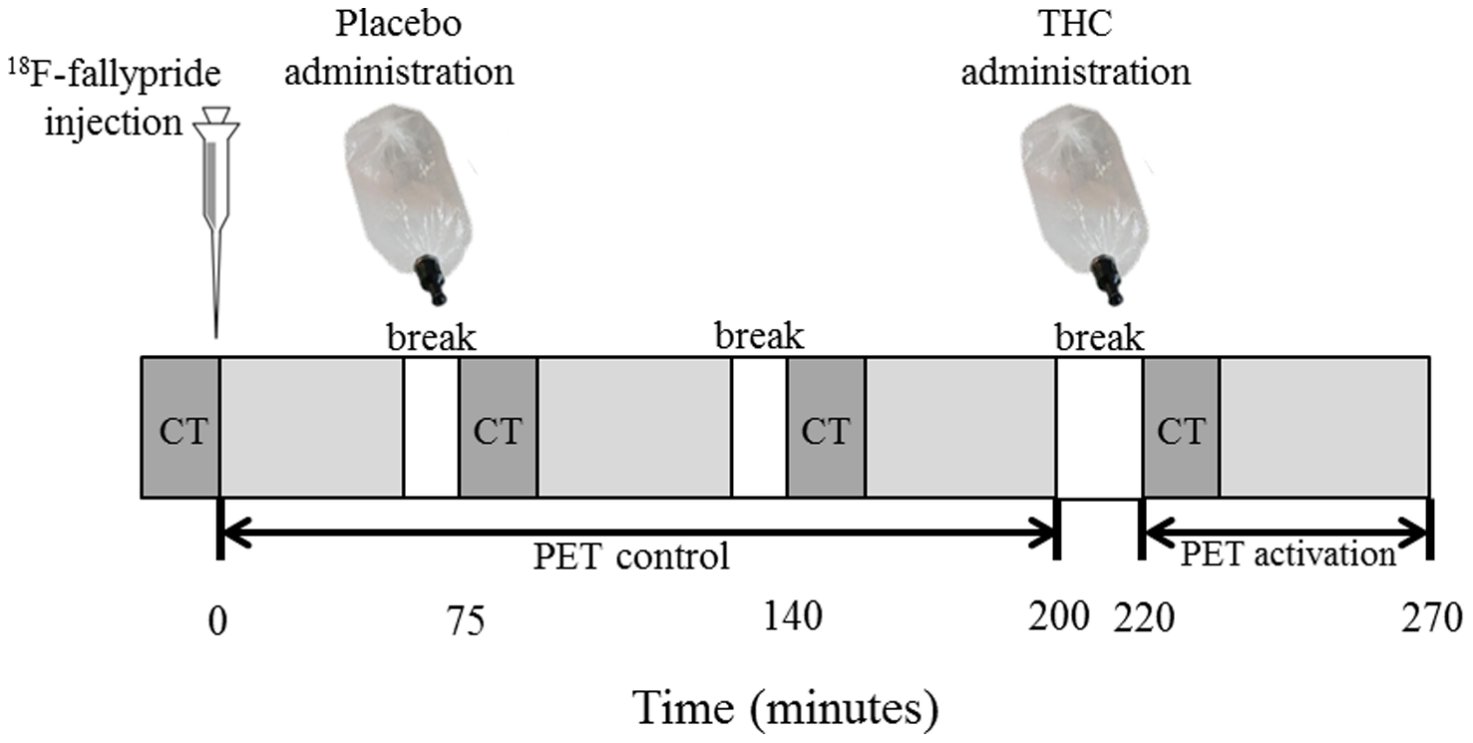
PET emission protocol conform the one-day PET imaging protocol for ^18^F-fallypride described and used previously by Christian and colleagues [34], modified according to simulation studies showing possible improvements in the experiment design that can increase the detection sensitivity of DA release in the striatum [37].

Subjects were placed on the scanner bed with their head restraint using a vacuum cushion and the body strapped to the bed to avoid movement during PET acquisition. Positions of the monitor and response box were adjusted to allow for optimal comfort. Subjects received on average 185 MBq of ^18^F-fallypride in a slow intravenous 10-second bolus injection through a catheter in the left antecubital vein. Mean injected dose was 187.4±8.7 MBq for controls, 190.5±7.0 MBq for relatives, and 189.4±5.0 MBq for patients. Upon tracer injection, dynamic emission scans were initiated in three-dimensional mode (3D) using a PET/CT scanner (Philips, Eindhoven, The Netherlands). Emission data were acquired in frames of 60 seconds during the first 6 minutes and in frames of 120 seconds thereafter. PET emission protocol was based on a previously reported one-day ^18^F-fallypride PET imaging protocol [34], [36], modified according to simulation studies showing possible improvements in the experiment design that can increase the detection sensitivity of DA release in the striatum [37] (see figure 1).

Emission data were collected in four segments (total scan duration excluding breaks was 220 minutes). Given the use of an “activation” parameter in the kinetic model used for analysis [38], representing presence of significant increase in the rate of ^18^F-fallypride displacement induced by the stimulus, and the hypothesis of Δ^9^-THC administration being associated with increased DA activity, the active (Δ^9^-THC) condition was always presented after the control (placebo) condition. The first three PET segments, with total scan duration of 170 minutes (separated by two brief breaks of 15 minutes) thus represented tracer kinetics during the control condition (the placebo was administered between the first and second PET segment). The control condition was followed by another break of 20 minutes upon which Δ^9^-THC was administered (i.e. at 220 minutes post-injection) and PET emission data were collected for another 50 minutes. The timing of Δ^9^-THC administration was chosen such that the radioligand binding would be at steady state conditions, and thus optimized for striatal brain regions according to a simulation study [37]. As can be concluded from Ceccarini et al. [37], stimulus administration at 200–220 minutes would result in positive gamma values around 0.000–0.002 for mid-range dopamine peak heights (i.e. around 200–245 nM), assuming that the real effect of Δ^9^-THC administration on dopamine release is in this range. To correct for attenuation, a low-dose (80 kV tube potential, 11 mA·s) CT scan without contrast agent was conducted at the beginning of each PET segment. Figure 1 illustrates the PET emission protocol.

Images were reconstructed using a 3D OSEM (ordered-subset expectation maximization) iterative reconstruction including model-based scatter as well as attenuation correction based on a measured attenuation map acquired by the CT, with a final spatial resolution of 4 mm. Additionally, in order to exclude structural brain abnormalities and perform anatomical coregistration, all subjects received a volumetric T1-weighted and standard transverse T2 brain magnetic resonance image (MRI; 1.5 Tesla Vision Scanner, Siemens, Germany). Parameters for the T1 3D Magnetization Prepared Rapid Acquisition Gradient Echo sequence were: TR = 0 ms, TE = 4 ms, flip angle = 12°, inversion time = 300 ms, matrix 256×256, 160 sagittal contiguous slices of 1 mm. For each subject, brain reconstructed PET data were transferred in DICOM (Digital Imaging and Communications in Medicine) and converted to Analyze using PMOD software v 2.95 (PMOD Inc., Zurich, Switzerland). To minimize effects of head movement during the scan, all ^18^F-fallypride frames for each PET scan were realigned, coregistered to the subject's MRI and then spatially normalized to a specific T1-weighted template constructed in MNI (Montreal Neurological Institute) stereotaxic space using SPM8 (Statistical Parametric Mapping, The Wellcome Department of Cognitive Neurology, London, UK). To increase signal to noise ratio, the normalized images were then smoothed with a 3D gaussian filter (4-mm full width at half maximum) before applying the kinetic model.

### Kinetic Modeling

Conform previous work [34], [35], [36], estimation of kinetic parameters representing Δ^9^-THC-induced DA release was performed by applying the linearized simplified reference region model (LSRRM) [38], an extension of the simplified reference region model (SRRM) [39], [40]. The LSRRM accounts for temporal perturbations in the ligand specific binding induced by pharmacological or non-pharmacological effects during a single-scan ^18^F-fallypride session including a baseline condition and an activation paradigm. The LSRRM assumes that a steady physiological state is disturbed throughout the experiment, by introducing a term γ· exp[-τ(t-T)] in the dissociation parameter (k_2a_ = k_2_/[1+BP_ND_]) of the simplified reference region model (SRRM), where k_2_ is the tissue to plasma efflux constant in the tissue region and BP_ND_ is the non-displaceable binding potential. γ represents the amplitude of the ligand displacement and the function h(t) describes a rapid change following activation onset and dissipation over time, where τ controls the rate at which activation effects die away; t denotes the measurement time and T is activation initiation time. The DA-radioligand competition at the D_2/3_ receptor sites is reflected by a temporal change of k_2a_ (via the change in BP_ND_), which is accounted for by a time-dependent parameter k_2a_+γ·h(t). Changes in BP_ND_ in activation studies are usually assumed to reflect changes in the concentration of available receptor sites, and a decrease in BP_ND_ is assumed to reflect increased neurotransmitter release. An increased k_2a_ therefore reflects a decreased BP_ND_ for D_2/3_ receptors, which will result in a positive value of γ. The cerebellum, representing an area with negligible density of D_2/3_ receptors, was used as reference region [39].

### Statistical Analysis

For each subject, two binary masks were created based on the corresponding normalized MRI, using an in-house created set of volumes-of-interests (VOIs) based on the Talairach atlas [41]. One binary mask contained all brain regions of interest (i.e. caudate nucleus, putamen, pallidum and nucleus accumbens), and a second mask was drawn only on the cerebellum. Regions of interest were chosen and restricted to the striatal regions based on Kuepper et al. [9], suggesting that the striatum is the primary area of interest for the convergence of Δ^9^-THC-effects on psychotic phenotypes, and Bossong et al. [19], providing first evidence for Δ^9^-THC-induced DA release in the striatum. For each subject, parametric maps of the receptor binding parameters (R [ = K_1_/K_1r_(reference region)], k_2_, k_2a_, BP_ND_ and γ) were computed. For each group, voxel-wise *t*-statistic maps of the γ parameters were computed over subjects to localize those areas with increased ligand displacement during Δ^9^-THC administration, thought to be proportional to an increased DA release. These statistic *t* maps were generated as *t* = γ/sd(γ), where the standard deviation parametric image of γ (sd[γ]) was created based on the estimated covariance matrix, consistent with previous work [34], [35]. The threshold of t was then set based on the degrees of freedom (df, df = n– p +1, with n = number of PET time points and p = parameter estimates) [34], with df = 110 for this work. A threshold of *t* >2.4 was used to represent *p*<0.01, for a one tailed *t* test. The relevant presence of activation-induced DA release was then presented by the percentage of significant voxels exceeding the threshold of *t* within each VOI. Additionally, a VOI analysis was performed by estimating the receptor binding parameters R, K_2_, K_2a_, BP_ND_ and γ using the LSRRM and the PET time-activity curves (TACs) over the VOIs. Group differences in the spatial extent of Δ^9^-THC-induced DA release were then tested using regression models within STATA. Inspection of residuals from the regression models indicated substantial heteroscedasticity of the error variances across the three groups. To account for this, we used a regression model that allowed the error variances to differ between groups.

Given hierarchical clustering of the behavioral (VAS) data, each person contributing more than one observation, VAS data were analyzed using multilevel random regression analysis in Stata using the XTREG routine, examining the effects of condition (placebo versus Δ^9^-THC) on subjective experience. Association between VAS scores and Δ^9^-THC-induced ligand displacement were analyzed using linear regression models with VAS scores as the dependent variable and Δ^9^-THC-induced ligand displacement as the independent variable. In addition, the interaction with group was calculated. Group differences in ligand displacement were analyzed using linear regression models with Δ^9^-THC-induced ligand displacement as the dependent variable and group as the independent variable. Although there were no suggestive differences between the three groups, these analyses were *a priori* adjusted for age, sex, nicotine use, alcohol use, use of other drugs and other medication and frequency of cannabis use.

## Results

### Participants

Two patients and one relative were excluded due to protocol violation in terms of use of antipsychotic medication and use of other drugs. In addition, two individuals (one relative and one control subject) were excluded due to excessive movement during the scan, yielding uncorrectable movement artifacts in the PET data. The resulting final sample thus consisted of 8 medication-free patients (2 medication-naïve, 4 medication-free for more than 3 years, 2 medication-free for minimally 10 days), 8 first-degree unrelated relatives and 9 healthy controls. Of the patients, five individuals fulfilled criteria for non-affective psychotic disorder and three individuals fulfilled criteria for affective psychotic disorder. There were no suggestive differences between the three groups with regard to mean age, male/female ratio, mean intellectual functioning as indexed by IQ, and frequency of cannabis use. Similarly, the groups did not differ with regard to current nicotine and alcohol use. Further, there were no differences with regard to injected dose of ^18^F-fallypride (*p*>0.05). Patients had higher scores on the positive syndrome dimension of the PANSS (see table 1 for demographic and clinical characteristics).

**Table 1:** Participant characteristics.

	Controls (n = 9)	Relatives (n = 8)	Patients (n = 8)
Mean Age (SD)	31.4 (11.4)	36.1 (12.0)	31.1 (8.9)
Percentage male (n)	55.6 (5)	62.5 (5)	75.0 (6)
Mean IQ (SD)	102.8 (14.0)	105.5 (11.8)	102.7 (15.6)
Frequency of cannabis use* % (n)			
* Monthly or less*	33.3 (3)	25.0 (2)	25.0 (2)
* Weekly*	11.1 (1)	12.5 (1)	12.5 (1)
* Daily*	55.6 (5)	62.5 (5)	62.5 (5)
Cannabis use frequency in heaviest period** % (n)			
* Monthly or less*	22.2 (2)	25.0 (2)	0.0 (0)
* Weekly*	11.1 (1)	12.5 (1)	12.5 (1)
* Daily*	66.7 (6)	62.5 (5)	87.5 (7)
Mean age of onset of cannabis use (SD)	16.8 (3.7)	14.9 (2.9)	15.4 (2.4)
Lifetime cannabis dependence*** % (n)	44.4 (4)	25.0 (2)	87.5 (7)
Current cannabis dependence*** % (n)	44.4 (4)	12.5 (1)	62.5 (5)
Weighted PANSS scores mean (SD)			
* Positive*	1.0 (0.0)	1.0 (0.1)	1.5 (0.7)
* Negative*	1.0 (0.0)	1.0 (0.1)	1.1 (0.2)
* Global*	1.2 (0.1)	1.1 (0.1)	1.2 (0.2)
* Total*	1.0 (0.0)	1.0 (0.1)	1.1 (0.1)
Nicotine use^a^ % (n)			
* 0*	44.4 (4)	25.0 (2)	25 (2)
* 1–10*	22.2 (2)	37.5 (3)	25 (2)
* 11–20*	33.3 (3)	37.5 (3)	37.5 (3)
* >20*	0.0 (0)	0.0 (0)	12.5 (1)
Alcohol use^b^ % (n)			
* 0–50*	66.7 (6)	50.0 (4)	62.5 (5)
* 50–150*	33.3 (3)	37.5 (3)	0.0 (0)
* 150* − *350*	0.0 (0)	12.5 (1)	37.5 (3)
Other medication use % (n)			
* Yes*	0.0 (0)	12.5 (1)	25.0 (2)
* No*	100.0 (9)	87.5 (7)	75.0 (6)
Use of contraceptives % (n)			
* Yes*	22.2 (2)	12.5 (1)	0.0 (0)
* No*	77.8 (7)	87.5 (7)	100.0 (8)
Use of other drugs^c^ % (n)			
* Yes*	44.4 (4)	37.5 (3)	37.5 (3)
* No*	55.5 (5)	62.5 (5)	62.5 (5)

*Note.* Percentages do not always total 100 due to rounding.

* Refers to cannabis use in the last 12 months.

** Refers to the frequency of cannabis used in the most intensive period of use as assessed with the CIDI.

*** Cannabis dependence according to the Structured Clinical Interview for DSM Disorders, *lifetime* and *current* (i.e. present in the last month).

a Refers to number of cigarettes per day.

b Refers to grams per week. Standard drink/unit size in the Netherlands contains 9.9 g of ethanol.

c Refers to other drug use in the last 12 months (incl. cocaine, opiates, psychostimulants, and sedatives).

### Drug Screening

Urinalysis was positive for Δ^9^-THC in 18 participants (6 patients, 6 relatives and 6 controls). Since the majority of the sample reported daily use of cannabis, urinalysis can be expected to reveal traceable amounts of Δ^9^-THC; all participants indicated compliance with the protocol and gave verbal confirmation of abstention from cannabis minimally 5 days before testing. All participants tested negative for alcohol or any of the other drugs.

### Blood Sample Analysis

The concentration of Δ^9^-THC in plasma reached a maximum of 37.3±19.3 ng/ml at 5 minutes post inhalation and decreased subsequently. The two main metabolites 11-OH-THC and THC-COOH reached a maximum concentration of 1.6±1.5 ng/ml and 25.2±22.4 ng/ml at 5 and 15 minutes post-inhalation, respectively. Plasma concentrations were not associated with group. Similarly, there was no difference in the baseline concentration of Δ^9^-THC, 11-OH-THC and THC-COOH in plasma between the three groups.

### Visual Analogue Scales

From the 13 VAS composite scores on ‘external perception’ (5 scales) and ‘internal perception’ (7 scales) were calculated. The scale on ‘feeling high’ was analyzed separately. As expected, Δ^9^-THC induced significant increases in ‘feeling high’ (β = 11.74, 95% CI: 6.90–16.59, *p*<0.001), ‘external perception’ (β = 2.16, 95% CI: 0.84–3.47, *p* = 0.001) and ‘internal perception’ (β = 1.19, 95% CI: 0.01–2.38, *p* = 0.049). There was no evidence for interaction between condition (placebo versus Δ^9^-THC) and group (controls, relatives and patients, all *p*>0.05), indicating that the effects of Δ^9^-THC on subjective experience were comparable across groups.

### In vivo DA Release in Response to Δ^9^-THC Administration

**Figure 2:**
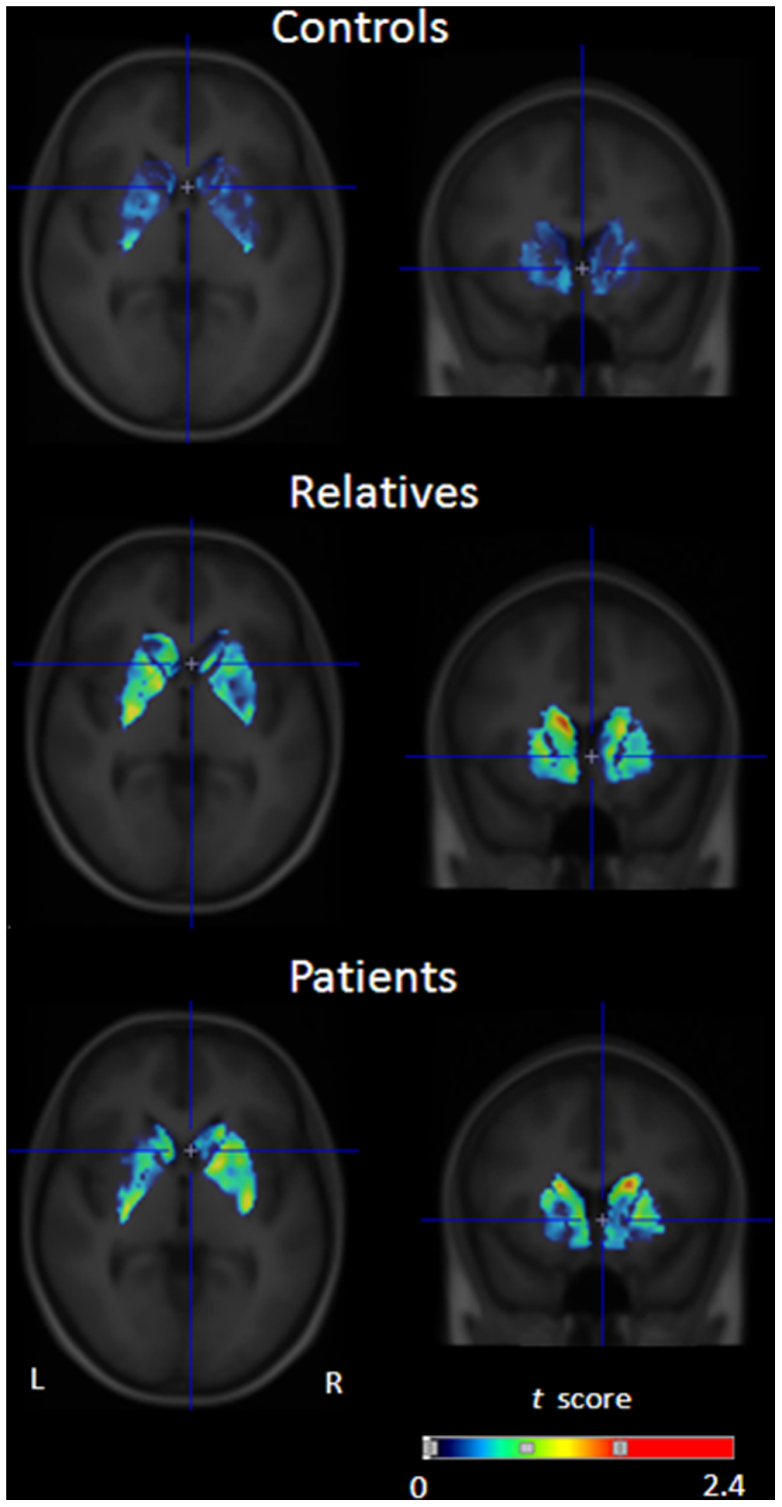
Mean statistical parametric *t* map of γ in sagittal (left) and coronal (right) sections overlaid on a MRI template, showing Δ^9^-THC-induced ^18^F-fallypride displacement at the level of the striatum (x = 0, y = 11, z = −4) for controls (top row, n = 9), relatives (middle row, n = 8) and patients (bottom row, n = 7, one subject was excluded from this analysis due to anomalous (non)physiological values). The image is thresholded for visualization purposes (maximum t obtained for controls, relatives and patients, respectively: 1.15, 2.6, and 2.7).

**Figure 3:**
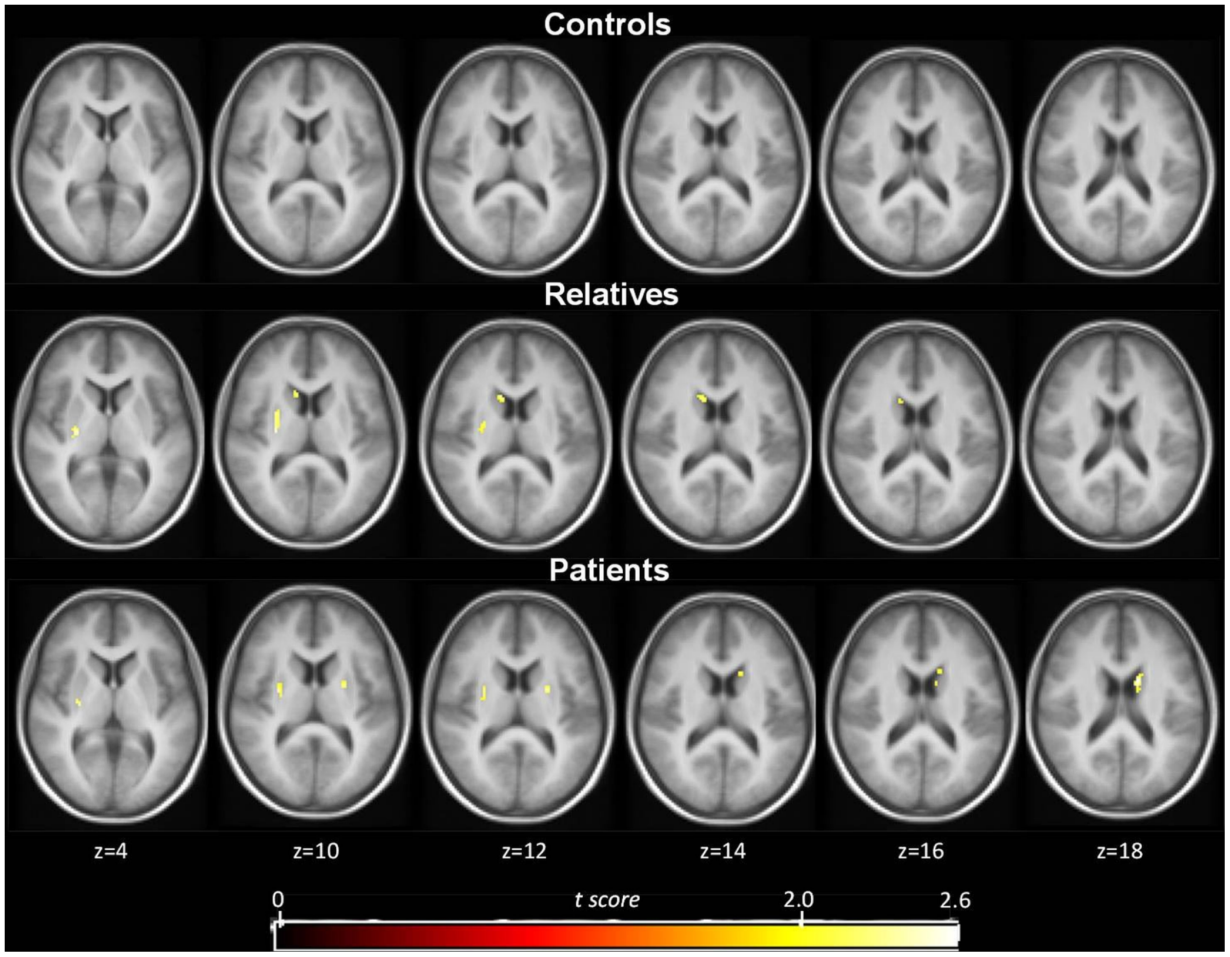
Mean statistical parametric t map of γ showing only the striatal voxels that show significant dopamine release surviving the t >2.4 threshold (p<0.01) in controls (top row, n = 9), relatives (middle row, n = 8) and patients (bottom row, n = 7). T maps are shown at the level of t >2.4 in transversal sections overlaid on a MRI template.

**Figure 4:**
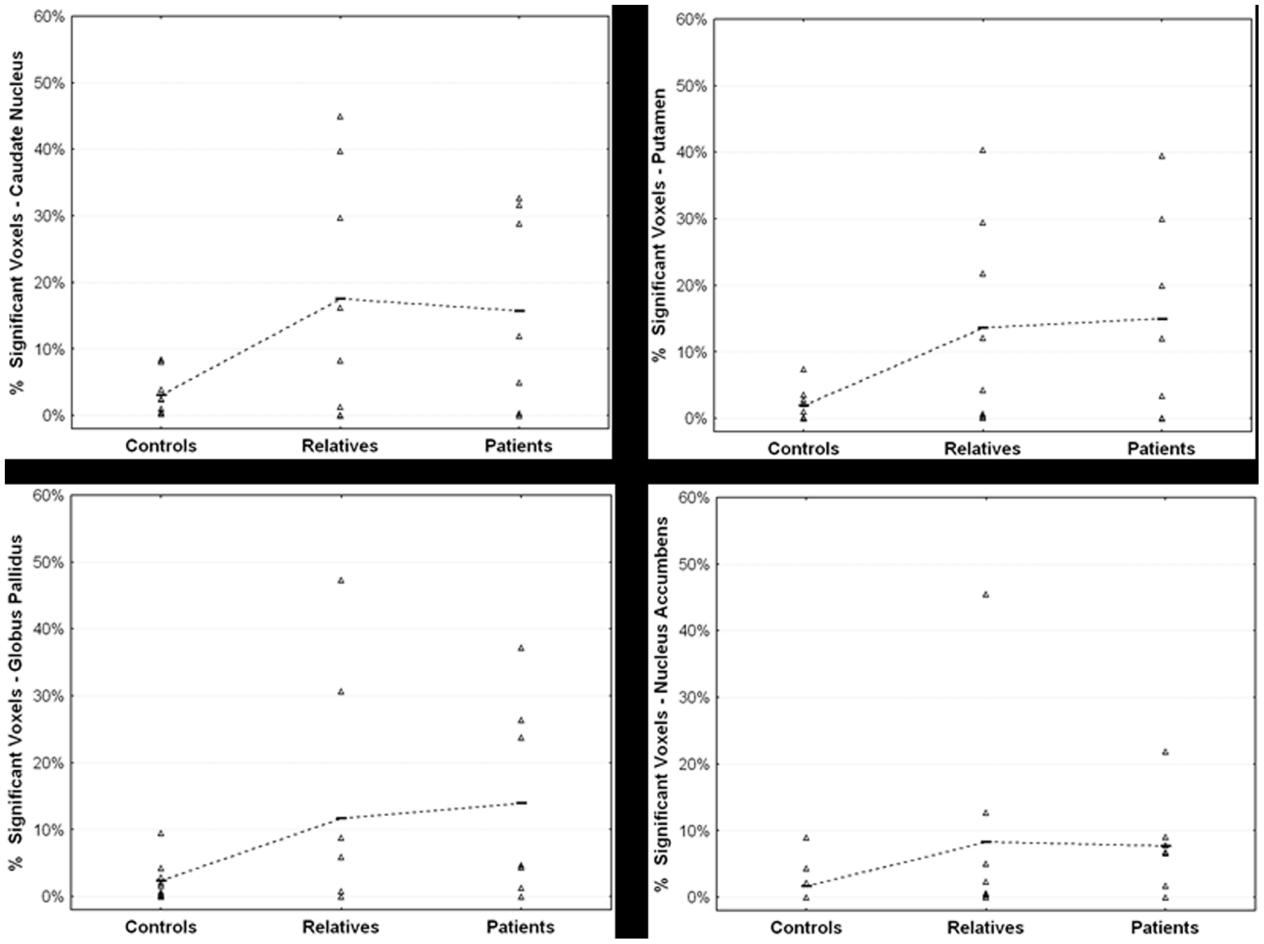
Percentage of voxels with significant Δ^9^-THC-induced dopamine release in the caudate nucleus, putamen (top row), globus pallidus and nucleus accumbens (bottom row) for controls (n = 9), relatives (n = 8) and patients (n = 7, one subject was excluded from this analysis due to anomalous (non)physiological values). Horizontal lines indicate the mean value for each group.

Due to methodological anomalies in form of non-physiological values, one subject (a relative) was excluded from the analyses. Δ^9^-THC induced significant ^18^F-fallypride displacement, indicative of DA release, throughout the striatum in both patients and relatives, but not in controls (see figures 2, 3 and 4, and table 2). On average, γ was positive (indicating DA release) for all striatal subregions in the patient group and for the caudate nucleus in the relatives (see table 3 for mean estimates of the kinetic parameters). Significant differences between the three groups with regard to the amount of ligand displacement were found in right and left caudate nucleus, left putamen, and right pallidum. However, only the difference in left caudate nucleus survived Bonferroni correction (see table 2 for group statistics). *Post hoc* pairwise comparisons showed that the amount of ligand displacement was significantly larger for patients and relatives versus controls, respectively, in left caudate nucleus (B_patients_ = 0.18, *p* = 0.002, B_relatives_ = 0.18, *p* = 0.021). No difference was observed between patients and relatives in this subregion. Group differences in ligand displacement were independent of age, gender, alcohol use, nicotine use, use of other drugs and other medication, and frequency of cannabis use.

**Table 2:** Spatial extent of estimated dopamine release induced by THC within striatal subregions.

	Controls (n = 9)	Relatives (n = 7^a^)	Patients (n = 8)	Group statistics#
***Caudate Nucleus***				
Right	3.5 (5.6)	13.7 (20.0)	15.3 (19.7)	B = 0.10; *p* = 0.011
Left	2.5 (3.0)	21.3 (19.6)	16.2 (16.9)	B = 0.12; *p* = 0.001*
Average	3.0 (3.2)	17.5 (18.3)	15.9 (15.1)	B = 0.11; *p* = 0.001
***Putamen***				
Right	1.4 (1.5)	10.8 (18.2)	16.2 (19.8)	B = 0.07; *p = *0.065
Left	2.4 (4.3)	16.4 (16.9)	13.7 (12.7)	B = 0.08; *p* = 0.008
Average	1.9 (2.5)	13.7 (15.3)	14.9 (15.5)	B = 0.08; *p* = 0.011
***Pallidum***				
Right	1.5 (2.7)	5.7 (7.3)	16.4 (21.8)	B = 0.07*; p* = 0.008
Left	3.3 (5.6)	17.6 (29.2)	11.6 (17.1)	B = 0.02; *p* = 0.671
Average	2.3 (2.8)	11.0 (16.6)	14.4 (15.6)	B = 0.07; *p* = 0.029
***N. Accumbens***				
Right	2.2 (5.7)	3.8 (7.7)	10.9 (16.1)	B = 0.04; *p* = 0.191
Left	1.2 (2.9)	12.8 (23.5)	4.5 (6.4)	B = 0.02; *p* = 0.109
Average	1.7 (3.1)	8.4 (15.6)	7.7 (7.1)	B = 0.04; *p* = 0.027
***Whole Striatum***	2.3 (2.0)	12.6 (13.8)	13.2 (12.0)	B = 0.07; *p* = 0.003

*Note.* Numbers are percentages (SD) of voxels within a region exceeding the significance threshold of *t* >2.4, relative to the total number of voxels within the mask of the respective region.

a One subject was excluded from this analysis due to anomalous (non)physiological values.

# Adjusted for age, gender, alcohol use, nicotine use, other drug use, use of other medication, and frequency of cannabis use.

* Significant after Bonferroni correction for multiple comparisons (i.e. at a statistical significance level of *p* = 1/8*0.05).

**Table 3:** Mean parameter estimates per region of interest (average left/right) and group.

	BP_ND_ (SD)	R (SD)	k_2_ (SD)	k_2a_ (SD)	γ (SD)	SSE (SD)
***Controls (n = 9)***						
Caudate nucleus	14.41 (2.59)	1.49 (0.37)	0.16 (0.02)	0.011 (0.002)	−0.0019 (0.0014)	0.67 (0.82)
Putamen	18.97 (4.06)	1.70 (0.37)	0.19 (0.02)	0.010 (0.002)	−0.0015 (0.0010)	0.51 (0.49)
Pallidum	12.03 (2.05)	1.30 (0.25)	0.12 (0.01)	0.010 (0.002)	−0.0014 (0.0019)	0.49 (0.47)
N. Accumbens	13.88 (2.58)	1.56 (0.36)	0.16 (0.03)	0.011 (0.002)	−0.0025 (0.0025)	0.98 (1.13)
***Relatives (n = 7*** a ***)***						
Caudate nucleus	13.32 (3.98)	1.29 (0.21)	0.15 (0.03)	0.012 (0.004)	0.0004 (0.0017)	0.60 (0.41)
Putamen	17.99 (4.62)	1.42 (0.21)	0.18 (0.03)	0.010 (0.003)	0.0000 (0.0020)	0.50 (0.19)
Pallidum	9.24 (3.18)	1.08 (0.14)	0.11 (0.02)	0.012 (0.004)	−0.0004 (0.0034)	0.56 (0.36)
N. Accumbens	13.18 (3.08)	1.25 (0.19)	0.15 (0.02)	0.011 (0.003)	−0.0021 (0.0042)	0.74 (0.28)
***Patients (n = 8)***						
Caudate nucleus	11.85 (3.88)	1.49 (0.34)	0.12 (0.03)	0.010 (0.002)	0.0009 (0.0034)	0.72 (0.64)
Putamen	16.79 (5.03)	1.74 (0.45)	0.16 (0.03)	0.009 (0.002)	0.0003 (0.0030)	0.89 (0.61)
Pallidum	10.89 (1.74)	1.40 (0.42)	0.11 (0.03)	0.010 (0.002)	0.0002 (0.0022)	0.87 (0.66)
N. Accumbens	10.13 (5.67)	1.44 (0.40)	0.11 (0.05)	0.009 (0.005)	0.0001 (0.0049)	2.02 (1.38)

a One subject was excluded from this analysis due to anomalous (non)physiological values.

No associations were found between Δ^9^-THC-induced changes on the VAS and Δ^9^-THC-induced DA release (all *p*>0.05).

## Discussion

The present study revealed the novel finding of differential striatal DA release following inhalation of Δ^9^-THC in individuals displaying different levels of psychosis risk. Patients with psychotic disorder and unaffected relatives released significantly more DA in response to THC compared to controls, who, consistent with most previous work, did not release significant amounts of DA.

### Δ^9^-THC-induced Dopamine Release: the Mechanism Behind Cannabis-induced Psychosis?

Numerous animal studies suggest that exogenous cannabinoids such as Δ^9^-THC stimulate burst firing of midbrain DA neurons and, as a consequence, facilitate striatal DA release through activation of CB1Rs, e.g. [16], [17]. In humans however, evidence that DA may mediate acute effects of Δ^9^-THC is scarce, and whether or not DA mediates in part the psychotogenic effects of cannabis remains unclear [9]. The results of previous imaging studies investigating acute Δ^9^-THC-induced striatal dopamine release in healthy men are inconsistent. While Bossong and colleagues [19] report a small, but significant increase in striatal DA, more recent work by Stokes and colleagues [20] as well as Barkus and colleagues [21] did not observe such an effect. Notably, Δ^9^-THC consistently induced changes in subjective perception and in scores on the BPRS (Brief Psychiatric Rating Scale) [42] or the PANSS (Positive and Negative Syndrome Scale) [26]. Yet, in none of the previous studies, the behavioral and psychotomimetic changes seen after Δ^9^-THC administration were associated with DA response. The lack of an association between subjective changes in perception as measured by the VAS and amount of DA release in the present study is thus in agreement with previous findings. Contrary though to what has been demonstrated previously [3], [4], [6], patients and relatives did not show evidence for increased sensitivity to the effects of cannabis in the current study as no differences in subjective perceptional changes associated with Δ^9^-THC were detected between the groups. Still, increased sensitivity was apparent on DA level as in both patients and unaffected relatives but not in healthy controls administration of Δ^9^-THC was associated with subsequent striatal DA release. Notably, in both patients and relatives, DA release was most pronounced in the caudate nucleus, and dopaminergic hyperactivity in this particular region is thought to play an important role in the pathophysiology of psychotic symptoms [43].

The present findings fit with animal work demonstrating interaction between the endocannabinoid and the dopaminergic system, in particular with regard to regulation of mesolimbic DA transmission. However, it has also been shown that part of the signaling activity mediated by the endocannabinoid system actually takes place downstream of DA neurotransmission in terms of D_2_ receptor activation, and DA may conversely regulate endocannabinoid function [44]. In line with this, elevated levels of the endocannabinoid anandamide in cerebrospinal fluid (CSF) were found in antipsychotic-naïve patients with acute psychosis, which is thought to be characterized by dopaminergic hyperactivity in striatal brain regions. The same elevation was found in patients treated with atypical antipsychotics, but absent in those treated with typical antipsychotics [45]. Moreover, elevated levels of anandamide may be present in patients in the prodromal phase of psychotic disorder [46]. In addition, a recent PET study has revealed elevated CB1R binding in schizophrenia patients [47].

Together these observations suggest an important role of the endocannabinoid system in the pathophysiology of schizophrenia and may furthermore explain our finding that exogenous cannabinoids such as Δ^9^-THC affect DA neurotransmission particularly in individuals at risk for DA dysregulation, such as patients with psychotic disorder and first degree relatives. Although not directly linked to the expression of psychotomimetic symptoms, DA might thus be involved in the increased risk of developing psychotic disorder associated with cannabis use in individuals with predisposition for psychosis.

### Limitations

Some limitations have to be acknowledged. The use of the LSRRM has several practical advantages, such as the requirement for only a single radiochemical synthesis and administration and avoidance of session effects. Additionally, since the model generates voxel-wise parametric calculations of the time-dependent kinetic parameters, it allows direct comparisons of DA release between subjects populations within a specific region if interest. However, practical implementation of the model implies that possible alterations in regional cerebral blood flow (rCBF) are not fully accounted for. Δ^9^-THC administration has been shown to be associated with bilateral rCBF increase, that is typically in the range of 5–15% [48], [49], although a ^15^O-water PET study found no significant rCBF change in the nucleus accumbens or other reward-related brain regions, nor in basal ganglia or hippocampus [50]. Simulation studies suggested that changes in rCBF around 25% could affect the outcome measure [34], [38], [51]. Therefore, even considering potential confound of rCBF induced by Δ^9^-THC, it is unlikely that these rCBF-related changes would add major perturbations in ligand displacement after Δ^9^-THC inhalation, representing consequently a possible confounding factor or bias for the outcome measure. In addition, as argued by Christian and colleagues [34], using a single injection protocol in combination with the in vivo kinetics of ^18^F-fallypride may minimize the possible confounds of changing rCBF associated with drug administration. Moreover, although schizophrenia patients might differ from healthy controls in both baseline as well as task-induced changes in rCBF, there is no evidence that THC differentially affects rCBF in healthy controls and unaffected first-degree relatives. Similarly, the model does not allow making inferences about baseline DA release capacity in the three groups. Yet, since the model estimates DA release associated with the active condition relative to the control condition, potential differences in baseline DA between the groups are indirectly accounted for.

Second, the spatial extent of the estimated Δ^9^-THC-induced DA release was obtained using a threshold of t >2.4 to represent p<0.01 (one-tailed t test) according to Christian et al. [34], yet without correction for the exact number of voxels included in the mask. Running the analyses with a corrected threshold of t >4.4 (i.e. corresponding to p<0.000012) expectedly results in markedly lower spatial extent of estimated DA release (for the caudate nucleus this was on average 0.1% for the controls, 1.6% for the relatives and 5.4% for the patients). However, the overall picture of the results remains unchanged (i.e. no DA release in the controls but in both relatives and patients).

Third, due to the constraint of a one-day protocol, the order of drug administration was single-blind and not random. However, since individuals were told that the order of administration would occur randomly, expectation bias seems unlikely and would not explain differential effects across the three groups. Further, compliance with the study protocol (i.e. abstinence from cannabis during the 5 days prior to scanning, abstinence from nicotine 4 hours prior to testing) could only be confirmed by interview and urinalysis was positive for 18 participants (75%). Yet, this is not surprising given that our sample included frequent cannabis users, of whom the majority used daily. Still, since baseline plasma levels of Δ^9^-THC and its main metabolites did not differ between the groups, it is unlikely that results were driven by differences in residual Δ^9^-THC. Concerning the use of nicotine, since the average amount of cigarettes used per day was equal across groups, this is unlikely to have influenced the PET outcome measure differentially across groups. Findings are therefore unlikely to have been biased by nicotine use. Fifth, it might be hypothesized that differences in cannabis use frequency between individuals could impact on the acute effects of Δ^9^-THC. However, while within groups there was indeed variability in cannabis use frequency there were no differences in cannabis use frequency between groups. In addition, cannabis use frequency was not associated with Δ^9^-THC-induced DA release in our current sample. Another issue that could limit the interpretation of our current results concerns past use of neuroleptic medication in the patient group. Only two patients were medication-naïve and increased sensitivity to Δ^9^-THC might be related to past use of neuroleptics. However, our current study demonstrates increased sensitivity to Δ^9^-THC not only in the patient group but also in unaffected relatives of patients with psychotic disorder, in whom we can exclude the influence of illness-related factors such as neuroleptic medication. Therefore, it appears unlikely that the observed effects in the patient group are explained by exposure to neuroleptic medication. Further, since we lack blood samples for tracer quantification, and there are no existing data on the effects of Δ^9^-THC on fallypride metabolism in healthy individuals compared to patients with psychotic disorder, we cannot rule out the hypothesis that differences in fallypride metabolism between the groups induced by Δ^9^-THC might have contributed to the current results. Finally, the present finding of a group difference in ligand displacement in several subregions of the striatum has to be interpreted in light of rather low power (0.6). Replication in a larger group is therefore imperative.
